# Efficacy of a herbal mouthwash for management of periodontitis and radiation-induced mucositis – A consolidated report of two randomized controlled clinical trials

**DOI:** 10.1016/j.jaim.2023.100791

**Published:** 2023-10-27

**Authors:** R. Ambili, K. Ramadas, Lekha M. Nair, Divya Raj, Farida Nazeer, Preethi Sara George, R. Rejnish Kumar, M. Radhakrishna Pillai

**Affiliations:** a Department of Periodontics, PMS College of Dental Science and Research, Thiruvananthapuram, India; b Department of Radiation Oncology, Regional Cancer Centre, Thiruvananthapuram, India; c Division of Dental Care, Regional Cancer Centre, Thiruvananthapuram, India; d Division of Cancer Epidemiology, Regional Cancer Centre, Thiruvananthapuram, India; e Rajiv Gandhi Centre for Biotechnology, Thiruvananthapuram, India

**Keywords:** Mouthwash, Herbal medicine, Periodontal disease, Mucositis

## Abstract

**Background:**

Oral diseases like periodontitis and mucositis often require home care using topical agents in the form of mouthwashes. Many herbal mouthwashes are found to be beneficial; however lack proper scientific evidence to support their use.

**Objectives:**

Study 1 evaluated clinical efficacy of herbal mouthwash in the management of chronic periodontitis in comparison with chlorhexidine mouthwash. Study 2 aimed at assessment of herbal mouthwash in patients of radiation-induced mucosititis.

**Methods:**

The novel herbal mouthwash used in the present study wa prepared from extracts of five plants namely *Emblica Officinalis, Terminalia chebula, Terminalia bellerica, Glycyrrhiza glabra,* and *Azadirachta indica*. 50 periodontitis patients were randomly allocated to two groups. As per allocation, they were instructed to use either herbal mouthwash or chlorhexidine mouthwash twice daily for two weeks after nonsurgical periodontal therapy. Similarly, patients with radiation-induced mucositis were randomly given herbal mouthwash and soda saline mouthwash. Intergroup and intragroup comparisons of continuous variables were conducted using paired and unpaired t-tests. Categorical variables were compared using the chi-square test.

**Results:**

Significant reductions in gingival bleeding, plaque accumulation, and pocket depth were noticed in periodontitis patients in both groups. Patients reported acceptable taste, freshness, and satisfaction after the use of herbal mouthwash. The herbal mouthwash group noticed a significant reduction in the severity of radiation-induced mucositis and analgesic requirements. The intensity of pain, dryness of mouth, oral hygiene, and need for the use of antibiotic and antifungal during radiotherapy was not significant among the groups.

**Conclusion:**

The results of this preliminary clinical trial support the use of the tested herbal formulation mouthwash as an adjunct in the treatment of periodontitis as well as radiation-induced mucositis.

**Clinical trial registration Number:**

For Study 1: CTRI/2019/10/021574, Study 2: CTRI/2020/04/024851.

## 1 Introduction

### 1.1 Background and rationale

Oral diseases are a major public health problem globally affecting almost 3.5 billion people worldwide [1]. Periodontal disease is the commonest oral disease among humans and 1 billion patients are suffering from severe periodontal diseases. Periodontal disease is initiated by microorganisms in dental plaque, but host immune inflammatory reaction plays an important role in the progression of the disease. Mechanical plaque removal is the conventional method for periodontitis, but it is often insufficient in many cases which requires adjunct measures. Mouthwashes are the most common adjunct measures in plaque control and among the various ingredients chlorhexidine is considered as the gold standard [2]. However, it possesses many disadvantages like cytotoxicity, antimicrobial resistance, staining, etc. (especially after long-term use which necessitates the identification of an alternate mouthwash).

Herbal medicines and other natural compounds have gained wide popularity in recent years compared to synthetic compounds for managing many chronic inflammatory diseases. Approximately 50% of clinically used drugs approved by the Food and Drug Administration are directly derived from or inspired by natural products [3]. They possess many advantages and the most important one to mention is their minimal side effects [4]. Herbal-derived agents had been used for the management of oral diseases for a long [5]. Herbal mouthwashes may have an edge over chlorhexidine mouthwash, especially for prolonged administration due to fewer side effects and better anti-inflammatory properties [6].

Oral mucositis is the most severe and prevalent complication of radiotherapy of head and neck cancers [7]. Salivary gland dysfunction associated with radiotherapy to the head and neck region leads to a reduction in the salivary flow and a change in the intra-oral pH. Saliva forms a thick coating over the mucous membrane which can lead to bacterial overgrowth. Severe mucositis can lead to treatment interruptions or premature termination of therapy resulting in poor tumor control and survival. All patients undergoing radiotherapy to the oral cavity develop some degree of mucositis. The severity of mucositis is influenced by the volume of tissue irradiated, the condition of normal tissues prior to irradiation, the dose per fraction and total dose, the pattern of application, quality of radiation, concurrent use of chemotherapy, preexisting medical condition of the patient and other host-related factors [8,9]. Oral mucositis is thought to be a complex biological process, involving direct damage to the dividing cell of the oral epithelium with depletion of the basal epithelium. It is modulated by the immune and inflammatory process and super-added infections by the oral bacterial flora, especially the aerobic gram-negative bacteria. Prevention and management of radiation-induced mucositis are critical as they can influence treatment outcomes, especially if radiation schedules have to be interrupted. Maintaining good oral hygiene and frequently rinsing the mouth with saline and sodium bicarbonate solution is generally recommended for preventing oral mucositis. Although several molecules such as amifostine, benzydamine, calcium phosphate, hydrolytic enzymes, zinc sulfate, granulocyte colony-stimulating factor (G-CSF), honey, etc. have been tried in preventing or reducing the severity of radiation/chemotherapy-induced mucositis, the available evidence is not strong to suggest a favorable efficacy. This necessitates the need for developing new treatment strategies [10].

Most of the commercially available herbal mouthwashes contain single ingredients. But Ayurveda claims the advantage of combining compatible ingredients in the right proportion to maximize the benefits. The novel herbal mouthwash used in the present study was prepared from extracts from five plants namely gooseberry *(Emblica Officinalis),* black- or chebulic myrobalan *(Terminalia chebula),* baheda (*Terminalia bellerica),* licorice *(Glycyrrhiza glabra)* and neem *(Azadirachta indica)*. Three components of the composition (*Emblica Officinalis, T.erminalia* *bellerica, and T.erminalia* *chebula*) are together known in classical Ayurveda as “Triphala” which literally translates to “three fruits”. Triphala has antibacterial, antiseptic, and anti-inflammatory properties. Even though individual components of present mouth rinse had been proven efficacious, to the best of our knowledge, this is the first study reporting the beneficial effects of this particular combination for treating periodontitis and mucositis.

### 1.2 Objectives

The objective of the present study was to evaluate the clinical efficacy of herbal mouthwash in the management of chronic periodontitis in comparison with chlorhexidine mouthwash. The study also aimed at evaluating the usefulness of herbal mouthwash in comparison to standard mouth care using frequent soda saline mouthwash for minimizing oral mucositis in patients undergoing radiotherapy for Head & Neck Cancers.

## 2 Methods

The study was conducted in two clinical settings to evaluate the efficacy of the novel herbal mouthwash. The first study was conducted in periodontitis patients and the second one was conducted in oral cancer patients undergoing radiotherapy.

### 2.1 Trial design

The study was randomized controlled parallel group trial.

#### 2.1.1 Study 1

Study 1 was carried out in the Department of Periodontics at PMS College of Dental Science and Research, Thiruvananthapuram, Kerala State. We have conducted the study in compliance with the Declaration of Helsinki. Consolidated Standards of Reporting Trials (CONSORT) guidelines were adopted [11].

##### 2.1.1.1 Ethics approval and registration

Institutional Ethics Committee approval was obtained [IEC NO: PMS/IEC/2018[ER]01] and the study was registered on CTRI (CTRI/2019/10/021574) before recruiting patients.

##### 2.1.1.2 Participants

Periodontitis patients (stage III) [12] without any systemic disease alone were included in the study. The criteria for categorizing periodontitis into stage III was ≥5 mm CAL (clinical attachment loss) and the number of tooth losses was less than 5. Patients who had taken antibiotics or anti-inflammatory medication for the past 3 months and undergone treatment for periodontitis within the last 6 months were excluded. Similarly, pregnant, and lactating females, smokers, and orthodontic patients were also not included in the study.

##### 2.1.1.3 Procedure

Participants provided written informed consent before starting the study. Detailed clinical examination was conducted which included: full mouth plaque score [13] (FMPS), bleeding score [14] (FMBS), and modified staining index [15]. Periodontal evaluation comprising of attachment loss and probing pocket depth (PPD) on six sites per tooth was carried out using UNC-15 probe‡ by the principal investigator (AR). Intra-examiner variability was assessed using kappa statistics. The same investigator provided non-surgical periodontal therapy for the study subjects using an ultrasonic scaler^§^.

##### 2.1.1.4 Interventions

The Herbal mouthwash was prepared by Ceego Labs Pvt. Ltd, (Kodambakkam, Chennai, Tamil Nadu, India) according to the composition described in the patent (Indian Patent No. 350995). One group received the herbal mouthwash (HM) and the control group received 0.2% chlorhexidine mouthwash (CHX) (Hexidine, ICPA Health Products Ltd, Andheri East, Mumbai, Maharashtra, India). Patients were instructed to rinse with both types of mouthwash twice daily, 30 minutes after brushing, without rinsing or eating for 30 minutes, and to avoid flossing or chewing gum during the study period. A dilution of 5 ml of herbal mouthwash with 20 ml water was decided based on the previous study where the same contents were used in powder form. The amount of ingredients were selected by the current manufacturers to ensure the same concentration in the liquid form, and we followed the manufacturer's recommendations. 10 ml of chlorhexidine was diluted with an equal quantity of water.

##### 2.1.1.5 Outcomes

Re-evaluation was completed after 2 weeks, and the primary outcome assessed was FMPS and FMBS. Secondary outcomes were changes in PPD and CAL. Patient-reported parameters such as pain in gums, bleeding and pus discharge from gums, bad breath, and sensitivity were recorded using a facial expression scale with a 0–10 grade. Taste, freshness, and overall satisfaction were rated on post-operative evaluation on a 4-point rating scale as bad, okay, good, and very good.

##### 2.1.1.6 Sample size

Statistical formula n = 2σ^2^ (Z_α_ +Z_β_)^2^/δ^2^ was applied to calculate the sample size [σ - standard deviation, Zα –Type I error (Zα = 1.96 at 95% CI), Zβ- Type II error (Zβ = 1.645 at 90% power), δ = minimum expected difference between the means]. Substituting the values minimum sample size required was 21 in each group which was upsized to 25 anticipating loss to follow-up.

##### 2.1.1.7 Randomization, sequence generation, allocation concealment, and blinding

The block randomization method was employed to allocate an equal number of patients to the HM and CHX group. Mouthwashes were given to patients by the clinical assistant based on allocations kept in a sealed opaque envelope. The outcome assessor was blinded to the allocation which was prepared by the statistician.

**Fig. 1:**
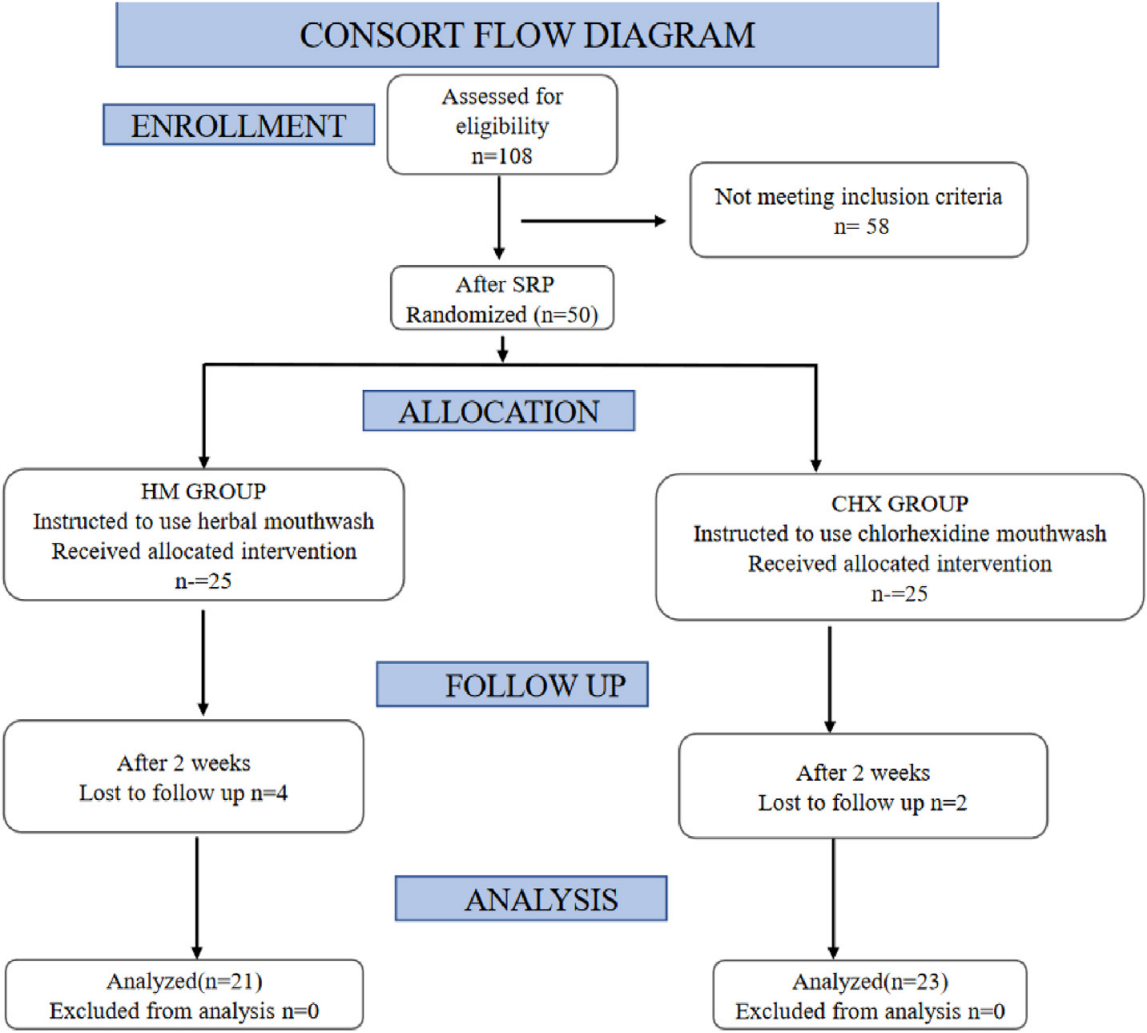
CONSORT Flow chart of design of Study 1.

A compliance diary was given to all participants to mark after each usage, and they were asked to return it along with empty bottles during reevaluation to ensure compliance. The study summary is given in Fig. 1.

#### 2.1.2 Study 2

##### 2.1.2.1 Participants

A total of 26 patients with oral cancer undergoing radical/adjuvant post-operative radiotherapy at the Regional Cancer Centre, Trivandrum were randomized into three groups after getting their informed consent. Patients requiring adjuvant chemoradiation were excluded. However, those who received chemotherapy before surgery were considered.

##### 2.1.2.2 Ethics approval and registration

Institutional Review Board (IRB No. 09/2019/13) and the Independent Human Ethics Committee (HEC No.39/2019) approved the study and was registered (CTRI/2020/04/024851).

##### 2.1.2.3 Interventions

26 patients were randomly allocated to three groups. 10 patients were advised to use the standard mouth care using frequent soda saline mouthwash (SS group). Five patients were advised to use the herbal powder mouthwash prepared by dissolving 30 gm of powder in 300 ml of water along with the usual mouth care (HP group). 11 patients were advised to use mouthwash prepared by dissolving 5 ml of liquid form of mouthwash dissolved in 20 ml of water (HM group). All the patients were advised to use the mouthwash four times daily during the entire course of radiotherapy. Patients were given radiotherapy 60Gy 30 fractions over six weeks’ time using the 2D or Conformal technique.

##### 2.1.2.4 Outcomes

**Fig. 2:**
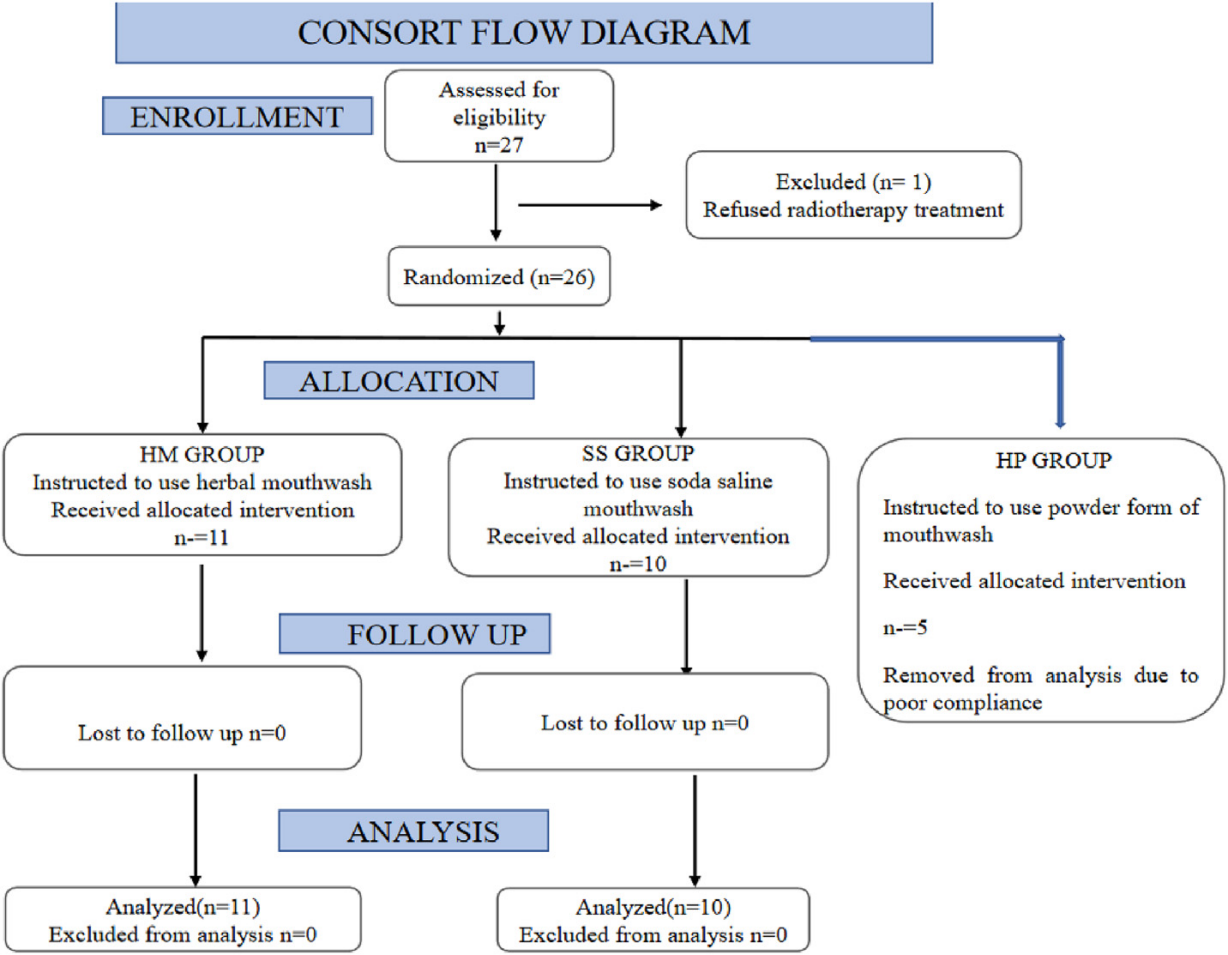
CONSORT Flow chart of design of Study 2.

All these patients were evaluated weekly by physicians, who were not aware of the randomization allotment. The mucositis grade was expressed on a 5-point scale using Radiation Therapy Oncology Group (RTOG) scoring system [16]. Grade I(M1): erythema and mild painful mucositis requiring no analgesics; Grade II(M2): patchy mucositis requiring analgesics; Grade III((M3): confluent mucositis and severe pain requiring narcotic analgesics; and Grade IV: deep ulcerations and/or necrosis (and sometimes bleeding), with extreme pain, and patients cannot eat anymore. The intensity of pain and dryness of mouth were scored on a 10 – point scale using a Visual Analog Scale (VAS) and were scored as mild (0–3), moderate (4–7), and severe (8–10). The use of analgesics, antibiotics and antifungal medications, and oral hygiene were also recorded in a structured proforma. The analgesic requirement was scored as per the WHO 3-tier system [17]. Measurements of Weight (Wt) were done before and after radiotherapy. Oral hygiene was assessed using Oral Hygiene Index (OHI) [18]. The study summary is given in Fig. 2.

##### 2.1.2.5 Sample size

The present trial was designed by assuming 80% power, 5% alpha level of significance (type 1 error), and the maximum difference in the mean mucositis scores between the two tests and the control arms was assumed as 1.1 from a pilot study, a total of 26 patients recruited.

##### 2.1.2.6 Randomization, sequence generation, allocation concealment, and blinding

Randomization into three groups was conducted using a computer-generated table by a statistician who was blinded. The outcome assessor was also blinded.

### 2.2 Statistical methods

The significances between the groups for normally distributed continuous variables (e.g., age) were tested using the independent t-test. Categorical variables were assessed using the Chi-square test. To assess the significance between two related samples (measured at two different time points), paired t-tests were used. A p-value <0.05 was considered significant. The compliance reported by use of ready-to-use powder form of the mouthwash was poor and was not considered for the analysis. All the statistical analyses were performed using SPSS, statistical software SPSS version 22.0 for WINDOWS.

## 3 Results

### 3.1 Study 1

Participants were recruited from November 2019 to March 2020. 21 patients in the HM group and 23 patients in the CHX group completed the study and they were considered for further analysis. The baseline characteristics of both groups were statistically comparable (Table 1).

**Table 1:** Baseline characteristics of participants in study 1 analyzed using independent test and chi-square test.

	HM Mean ± SD	CHX Mean ± SD	p-value
Age(years)	48.28 ± 11.79	51.04 ± 13.29	0.21^a^
Gender, n (%)			
Male	9(42.86)	10(43.48)	0.79^a^
Female	12(57.14)	13(56.52)	
PPD (mm)	3.71 ± 0.83	3.68 ± 0.56	0.94^a^
CAL (mm)	4.56 ± 0.82	4.28 ± 1.03	0.33^a^
FMPS %	79.2 ± 6.16	74.5 ± 12.76	0.13^a^
FMBS%	86.58 ± 14.76	89.44 ± 9.28	0.44^a^

PPD, probing pocket depth; CAL, clinical attachment loss; FMPS, full mouth plaque score; FMBS, full-mouth bleeding score; SD, standard deviation; HM, herbal mouthwash group; CHX, chlorhexidine group.

a Statistically not significant (p > 0.05).

#### 3.1.1 Outcome and estimation

Significant reduction in clinical parameters was noticed after 2 weeks in both groups (Table 2). Intergroup comparison of reduction in clinical parameters was statistically not significant (p < 0.05).

**Table 2:** Comparison of clinical parameters of test and control group in study 1, intergroup comparison using paired t-test.

		Stage	Mean	SD	n	P
FMBS (%)	HM	Baseline	79.2	6.16	21	p < 0.0001^a^
		After 2 weeks	35.97	5.64	21	
	CHX	Baseline	74.5	12.76	23	p < 0.0001^a^
		After 2 weeks	39.20	9.46	23	
FMPS (%)	HM	Baseline	89.58	14.76	21	p < 0.0001^a^
		After 2 weeks	46.75	8.14	21	
	CHX	Baseline	89.44	9.28	23	p < 0.0001^a^
		After 2 weeks	48.60	7.58	23	
PPD in mm	HM	Baseline	3.71	0.83	21	p < 0.0001^a^
		After 2 weeks	2.63	0.28	21	
	CHX	Baseline	3.68	0.56	23	p < 0.0001^a^
		After 2 weeks	2.92	0.80	23	
CAL in mm	HM	Baseline	4.56	0.82	21	p < 0.0001^a^
		After 2 weeks	3.19	0.68	21	
	CHX	Baseline	4.28	0.83	23	p < 0.0001^a^
		After 2 weeks	3.08	0.78	23	

PPD, probing pocket depth; CAL, clinical attachment loss; FMPS, full mouth plaque score; FMBS, full mouth bleeding score; SD, standard deviation;

HM, herbal mouthwash group; CHX, chlorhexidine group.

a Statistically significant (p < 0.05).

We then evaluated the patient-reported outcomes after the use of mouthwash. The results are presented in Table 3. Sixty percent of the patients reported a reduction in tooth sensitivity while 40% had a feeling of increased sensitivity after the use of herbal mouthwash. A statistically significant increase in sensitivity was noticed in the chlorhexidine group (68%). Most of the patients rated the taste of mouthwash as good and freshness and overall satisfaction was good to very good. Staining of teeth was not observed in any of the patients treated with herbal mouthwash.

**Table 3:** % reduction in patient-reported outcomes after the use of herbal mouthwash and chlorhexidine.

Item	CHX	HM
Gum pain	72%	76%
Pus discharge	70%	67%
Bleeding from gums	71%	75%
Bad breath	68%	76%

#### 3.1.2 Adverse events

No adverse events were reported in both the groups.

### 3.2 Study 2

A total of 26 patients were recruited into the study between November 2020 and April 2021. 10 patients were allotted to SS group, five patients to HP group and 11 patients to HM group. The compliance with the powder form of mouthwash was extremely poor and they were not considered for the analysis. The baseline characteristics were comparable and are shown in Table 4.

**Table 4:** Baseline Characteristics of patients included in study 2 analyzed using chi-square test, p < 0.05 - Statistically significant.

Variables		SS (10)	HM (11)	P Value
		n	n	
Gender	Male	8	6	0.135
	Female	2	5	
Hypertension	No	7	8	0.918
	Yes	3	3	
Diabetes	No	9	8	0.391
	Yes	1	3	
Tobacco chewing	Never	5	5	0.672
	Ever	5	6	
Alcohol	Never	6	6	0.239
	Ever	4	5	
Smoking	Never	7	7	0.126
	Ever	3	4	
Site of cancer	Tongue	6	3	0.452
	Buccal mucosa	2	2	
	Others	2	6	
Histology	WD	4	5	0.932
	MD	5	5	
	NOS	1	1	
T-Stage	T1	2	1	0.649
	T2	4	3	
	T3	3	2	
	T4	1	5	
N-Stage	N0	4	4	0.692
	N1	3	5	
	N2	3	1	
	N3	0	1	
Composite Stage	Stage 1	1	1	0.714
	Stage 2	2	1	
	Stage 3	2	3	
	Stage 4	5	6	
Chemotherapy	No	6	6	0.962
	Yes	4	5	
Surgery	No	0	1	0.492
	Yes	10	10	

All patients completed the full course of treatment. There was no interruption in radiotherapy treatment in both groups. No significant difference in mucositis was observed midway between the treatment (week 3). However, significant difference in mucositis was observed at the end of radiotherapy in patients in the HM group. Grade 3 mucositis was 63.6% vs 100% favoring intervention (p = 0.04; Table 5).

**Table 5:** Toxicities during and at the end of radiotherapy in both groups compared using chi square test, p < 0.05 -Statistically significant.

Variable	Time of evaluation		SS (n = 10)	HM (n = 11)	p value
Grade of mucositis	3rd Week	M1	0	2	0.259
		M2	6	4	
		M3	4	5	
	End of Radiotherapy	M2	0	4	0.043
		M3	10	7	
Intensity of pain	3rd Week	0	1	1	0.896
		STEP 1	5	6	
		STEP 2	3	4	
		STEP 3	1	0	
	End of Radiotherapy	0	1	0	0.387
		STEP 1	1	3	
		STEP 2	4	4	
		STEP 3	4	4	
Analgesic use	3rd Week	0	1	1	0.896
		STEP 1	5	6	
		STEP 2	3	4	
		STEP 3	1	0	
	End of Radiotherapy	0	1	0	0.387
		STEP 1	1	3	
		STEP 2	4	4	
		STEP 3	4	4	

M1: erythema and mild painful mucositis requiring no analgesics; M2: patchy mucositis requiring analgesics; M3: confluent mucositis and severe pain requiring narcotic analgesics.

Table 5 shows the intensity of pain after 3 weeks and at the end of radiotherapy. of treatment (3 weeks) 20% of patients in the SS group reported severe pain compared to none in the HM group though not significant. The intensity of pain was similar in both groups at the end of radiotherapy. However, analgesic use was less in the HM group at the end of radiotherapy. Twenty-seven percent of patients in the HM group had only step 1 analgesic compared to 10% in the SS group (Table 5).

Table 6 shows the intensity of dryness of mouth and oral hygiene before and after radiotherapy which were similar in both groups. Table 7 shows the use of antibiotics and antifungals during radiotherapy that was similar in both groups.

**Table 6:** Severity of dryness of mouth and oral hygiene before and at the end of radiotherapy analyzed using chi-square test, p < 0.05 -Statistically significant.

Variable	Time of evaluation		SS (n = 10)	HM (n = 11)	p value
Dryness of mouth	Before RT	No dryness	6	9	0.492
		Minimal	4	2	
	After RT	NA^a^	0	1	0.564
		Minimal	6	4	
		Moderate	4	3	
		Severe	0	3	
Oral hygiene	Before RT	Poor	1	1	0.263
		Fair	4	2	
		Good	5	8	
	After RT	NA	1	1	0.524
		Poor	0	2	
		Fair	4	4	
		Good	5	4	

a Did not report for evaluation at the end of radiotherapy.

**Table 7:** Antibiotic and Antifungal use during radiotherapy analyzed using chi-square test, p < 0.05 -Statistically significant.

Variables		SS (10)	HM (11)	P Value
		n	n	
Antibiotic use		0	0	0.276
	No	8	7	
	Yes	2	4	
Antifungal use		0	0	0.310
	No	7	9	
	Yes	3	2	

Two patients in the HM group had minimal discomfort using the mouthwash. All the patients reported weight loss. The mean change in weight before and after radiotherapy was 1.9 kg in the SS group and 1.6 kg in the HM group, which was not significant. Two patients each in both groups nasogastric tube feed at the time of initiation of radiation. One patient in the control arm and two patients in the intervention arm were given nasogastric tube insertion during treatment.

## 4 Discussion

Drugs based on natural products are rapidly gaining acceptance over synthetic chemical compounds for managing oral diseases like periodontal diseases and mucositis. The efficacy of herbal mouthwash for the management of two common inflammatory diseases of the oral cavity namely periodontitis and radiation-induced mucositis was evaluated in the present study.

Mouthwashes are widely used in the management of periodontitis. The disadvantages of many commercially available mouthwashes limit their long-term use. Ayurvedic formulations were used as oral rinses to manage periodontitis [19]. The ayurvedic composition used in the present study was patented (Indian Patent No. 350995) and was found effective in the management of radiation-induced oral mucositis in oral cancer patients in a previous study conducted [20]. The intensity of analgesic usage was also less in these patients. The same formulation was found to be effective as an adjunct to mechanical therapy in the management of periodontitis as well (unpublished data). The powder form of the ingredients was used in the above-mentioned studies where overnight soaking was required to get the supernatant which was used as a mouth rinse. To overcome the difficulties encountered by patients in the preparation of mouth rinse, the current study was conducted with mouthwash using the same ingredients in the same quantity in a ready-to-use liquid form. We could observe a statistically significant reduction in gingival inflammation, plaque accumulation, and pocket depth after adjunctive use of herbal mouthwash. Improvement in clinical parameters observed in our study after the use of herbal mouthwash was comparable to chlorhexidine.

The three components of the present composition form triphala which has proved useful for treating periodontal diseases and had been well-documented in multiple studies [21,22]. Triphala also inhibits PMN–type matrix metalloproteinase (MMP- 9) which is again of advantage in managing periodontitis [21]. Our results are in accordance with a randomized controlled clinical trial where Triphala mouthwash was found to be equally effective as chlorhexidine in reducing gingival inflammation, plaque accumulation, and microbial count in gingivitis patients [22].

Other components of the present composition also possess properties beneficial for the treatment of inflammatory diseases. Multiple clinical studies have reported improvement in clinical parameters after using *A. indica* mouthrinse [23]. Different parts of the plant have anti-inflammatory, antimicrobial and antioxidant properties [24]. Similarly, glycyrrhiza glabra is an antioxidant, anti-inflammatory, and antimicrobial agent [25]. Moreover, its sweet flavor will compensate for the bitter taste of other ingredients in the tested composition and can stimulate taste receptors which in turn will improve the salivary flow rate and reduce xerostomia for 3 hrs after the usage [26]. This is advantageous for treating radiation-induced mucositis as well as periodontitis and associated halitosis.

A similar composition comprising Tulsi, turmeric, Triphala, *A. indica*, honey, and mint Mentha leaves also provided better antimicrobial properties compared to chlorhexidine [27]. As observed in our study, many such herbal formulations are reported to be as effective as chlorhexidine [[28], [29], [30]]. Similar reports are published in a recent systematic review and meta-analysis [31]. Although chlorhexidine is considered a gold standard as a chemical plaque control agent, its long-term use is limited due to side effects like staining of teeth, taste disturbances, and cytotoxic effects on oral tissues [32,33]. Hence the natural herbal-based agents as employed in the present study are a promising alternative to chlorhexidine.

Patient-reported outcome after the use of herbal-based mouthwashes has rarely been reported in the literature. In the present study, patients reported a significant reduction in gum pain, pus discharge, bleeding from gums, and bad breath after the use of herbal mouthwash. Sensitivity is a common complaint of periodontal patients especially after scaling. The results of the present study indicate that our herbal mouthwash is effective in reducing post-scaling sensitivity. Most of the patients reported an initial bitter taste which later turned out to be freshness which may be because of gooseberry content *(Emblica Officinalis)*. None of the patients developed staining of teeth which is a very common finding after the use of chlorhexidine mouthwash. Moreover, a significant number of patients reported good to great overall satisfaction after using the herbal mouthwash.

In our second study, a significantly more effective reduction in the severity of mucositis was noticed compared to standard-of-care soda saline mouthwash. Similarly, the intensity of analgesic use was less in the herbal mouthwash group. However, the intensity of pain, oral hygiene, and dryness of mouth were similar in both groups. There were no adverse events reported and compliance with the liquid form of herbal mouthwash was good. Many previous clinical trials had reported similar observations after using other herbal formulations [34,35]. Shah et al. recently found out that 0.1% curcumin mouthwash significantly delayed the onset of radiation-induced mucositis. Even though fewer number patients developed mucositis in the curcumin group compared to the benzydamine group it was not statistically significant. A recent meta-analysis concluded that anti-inflammatory mouthwashes are useful for providing symptomatic relief for radiation-Induced oral mucositis [36]. The anti-inflammatory property of the ingredients used in the present formulation may be responsible for the clinical improvement noticed. *Glycyrrhiza glabra* can increase the salivary flow and in a previous report, it was found to be advantageous in reducing the severity of radiation mucositis compared to a placebo mouthrinse [37]. In our study, we could not detect a significant difference in the dryness of the mouth after using herbal mouthwash.

One of the main limitations of the study is the limited number of samples. Even though several clinical improvements were noted in the study, the basic mechanisms underlying these changes were not evaluated in the present study. Two studies were designed to test the efficacy of herbal mouthwash in minimizing inflammation and superadded infection in two different situations, radiation-induced oral mucositis and periodontitis, for a common purpose. Future clinical trials with a greater number of samples are required with supporting cell biology research to provide more evidence regarding the mechanism of action.

## 5 Conclusion

Within the limitations of the study, the tested herbal mouthwash was found to decrease the severity of radiation-induced mucositis and intensity of analgesic requirements, gingival inﬂammation, plaque accumulation, probing depth, and halitosis. Fewer side effects were noticed compared to commercially available mouthwashes. Hence the present herbal formulation is promising and can be considered a potential therapeutic agent in the treatment of periodontitis as well as for the prevention and treatment of radiation-induced mucositis.

## Author Contribution

**R. Ambili**: Conceptualization, Methodology, Study design, Formal analysis, Investigation, Data curation, Writing – original draft, Writing – review and editing, Supervision. **K. Ramadas:** Conceptualization, Methodology, Study design, Investigation, Data curation, Writing – original draft, Visualization, Supervision. **Lekha M. Nair:** Investigation, Data curation, Project administration. **Divya Raj:** Formal analysis, Investigation, Data curation, Writing – review and editing, Project administration. **Farida Nazeer:** Investigation, Data curation, Project administration. **Preethi Sara George:** Methodology, Study design, Validation, Formal analysis, Writing – review and editing. **R. Rejnish Kumar:** Formal analysis, Investigation, Writing – review and editing. **M. Radhakrishna Pillai:** Conceptualization, Methodology, Study design, Writing – review and editing, Visualization, Funding acquisition.

## Funding

No other source of funding for the study.

## Declaration of competing interest

None.
